# Caterpillar Venom: A Health Hazard of the 21st Century

**DOI:** 10.3390/biomedicines8060143

**Published:** 2020-05-30

**Authors:** Andrea Seldeslachts, Steve Peigneur, Jan Tytgat

**Affiliations:** Toxicology and Pharmacology, KU Leuven, Campus Gasthuisberg, O & N2, Herestraat 49, P.O. Box 922, 3000 Leuven, Belgium; andrea.seldeslachts@kuleuven.be (A.S.); steve.peigneur@kuleuven.be (S.P.)

**Keywords:** caterpillar venom, venomics, pathophysiology, antivenom, treatments

## Abstract

Caterpillar envenomation is a global health threat in the 21st century. Every direct or indirect contact with the urticating hairs of a caterpillar results in clinical manifestations ranging from local dermatitis symptoms to potentially life-threatening systemic effects. This is mainly due to the action of bioactive components in the venom that interfere with targets in the human body. The problem is that doctors are limited to relieve symptoms, since an effective treatment is still lacking. Only for *Lonomia* species an effective antivenom does exist. The health and economical damage are an underestimated problem and will be even more of a concern in the future. For some caterpillar species, the venom composition has been the subject of investigation, while for many others it remains unknown. Moreover, the targets involved in the pathophysiology are poorly understood. This review aims to give an overview of the knowledge we have today on the venom composition of different caterpillar species along with their pharmacological targets. Epidemiology, mode of action, clinical time course and treatments are also addressed. Finally, we briefly discuss the future perspectives that may open the doors for future research in the world of caterpillar toxins to find an adequate treatment.

## 1. Introduction

Envenomation by caterpillars constitutes an emerging public health issue of international concern. Caterpillars belong to the order Lepidoptera, the second largest order of insects in the phylum Arthropoda. This order comprises approximately 160,000 species organized in 43 superfamilies with 133 families, distributed around the globe [1]. Many species are recognized by their beautiful and colorful patterning scales and hairs (called setae or spines) on the body [2]. Of the 133 families, nine families (Erebidae, Eucliedae, Lasiocampidae, Limacodidae, Megalopygidae, Notodontidae, Nymphalidae, Saturniidae and Zygaenidae) cause severe pathophysiological conditions and are commonly involved in human and animal intoxications [3,4].

These accidents are reported throughout the world and, in some communities, this has even reached epidemic records which underscores the high burden [5]. Often, accidental contact involves a single caterpillar or, more severely, a colony with a dangerous number of caterpillars camouflaged in the trees [6]. However, in some countries, the reactions are even seen over a considerable distance from the origin. This is due to the capacity of some caterpillar species to release the setae into the air as part of their defense system, comparable with the problem caused by pollen. Contact with the airborne setae is sufficient to cause local and/or systemic reactions [7]. Among caterpillar species, the most dangerous species are classified within the family Saturniidae and are responsible for severe and fatal accidents, occurring mainly in the tropical climate zones. In these countries, caterpillar envenomation is relatively more common but still remains an underestimated problem with considerable health and economical damage.

Most of the health symptoms are consequences of a direct, usually under accidental circumstances, or indirect (air) contact with the urticating setae/spines. In some cases, the hemolymph or other droplets produced by the poisonous larvae of the caterpillar can have toxic properties [8,9]. This means that caterpillars can be both poisonous (via hemolymph or other droplets) and venomous (i.e., toxins delivered via setae or spines). These substances are used as a defense system and allow the caterpillar to respond actively against predators. For example, after physical irritation, the chitin-rich tips at the distal end of the setae readily break and release a complex venomous cocktail [10].

As a result of positive evolutionary pressure, this venomous cocktail offers a wide spectrum of biochemical and toxicological diversity that interferes with important physiological functions for humans and animals [11]. Despite multiple studies already being performed on the content and interaction of venom components of, e.g., snakes, conus, spiders and scorpions, caterpillar species have been somewhat neglected. The already investigated mixtures consist of components such as proteins, enzymes, peptides, allergens and/or low molecular molecules that determine a wide range of clinical manifestations. Depending on the family and species involved, some toxins provoke local urticating dermatitis, a burning sensation, allergic reactions, respiratory system problems and/or opthalmia nodosa, whereas others cause systemic effects, including hemorrhagic syndrome, acute kidney injury and/or phalangeal periarthritis [3].

With recent advances in cutting-edge technologies such as genomics, transcriptomics and proteomics, the venom of caterpillar species can be explored in unprecedented detail. In this way, a collection of diverse molecules involved in the pathology has been surmised. Despite state-of-the-art advancements in the qualitative and quantitative characterization of venom toxins, the target(s) of the venom component(s) in many species remain poorly understood. Hence, it is difficult to make a clear correlation between the identified components and the reactions seen. Additionally, for many species, the venom composition is still not well known and often displays a variability between species [12].

This review summarizes the main issues of caterpillar envenoming, including the epidemiology, mechanism of action and the diversity of molecules involved in the clinical manifestations. In this way, the review aims to enhance our knowledge of the already identified bioactive component(s), their role on human body target(s) and finally combine this information into an integrated framework for future research. The diagnosis and current treatments are highlighted, together with the future perspectives to better understand and confront caterpillar envenomation with a true selective medicine.

## 2. Evolving Global Epidemiology

**Figure 1:**
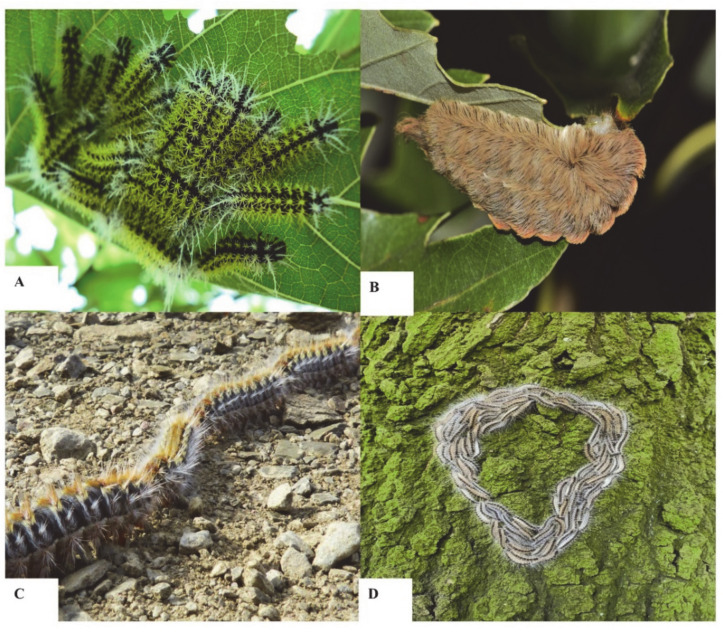
Representation of different venomous caterpillar species. (**A**) *Leucanella memusae*, (**B**) *Megalopyge opercularis*, (**C**) pine processionary caterpillar (*Thaumetopoea pityocampa*), (**D**) oak processionary caterpillar (*Thaumetopoea processionea*).

Every year, thousands of people worldwide are affected either after direct contact with a caterpillar or indirectly via air or objects on which a caterpillar has moved. In the 21st century, envenomation by caterpillars is still clinically challenging owing to their potential to provoke a diverse array of symptoms. Nowadays, doctors have limited means at their disposal to relieve symptoms caused by many caterpillar species. Treatments are mainly supportive and not efficient. The only specific treatment on the market is the *Lonomia* antivenom. It is used to address the intoxication by the extremely venomous *Lonomia obliqua* from the Saturniidae family, commonly known as Taturana or fire caterpillar, and predominantly found in southern Brazil (Table 1) [13]. Despite the introduction of an antivenom therapy in 1994, mortality rates due to *Lonomia* species continue to occur [14]. The high morbidity and lethality are mainly induced by the development of an acute kidney injury [15]. In Brazil from 2000 to 2018, the Ministry of Health reported 60,588 caterpillar envenomation cases, of which there were 33 mortalities, and an incidence rate of 3.2 envenomations per 100,000 inhabitants [16]. From the same family, Saturniidae, several cases of envenomation by *Hylesia metabus* and *Leucanella memusae* (Figure 1A) were also reported as indicated in Table 1 [17,18].

In the United States, *Megalopyge opercularis* from the family Megalopygidae, known as woolly slug or puss caterpillar, has gained a foothold (Figure 1B, Table 2) [19]. A recent study by the Texas Department of State Health Service described 3484 *M. opercularis* caterpillar envenomations reported by the Texas Poison centers between 2000 and 2016 [20]. Although envenomation by *M. opercularis* can occur throughout the year, the levels of envenomation reach their peak in July and in the period from October to November. In Asia, epidemic peaking outbreaks of *Dendrolimus pini* and *Euproctis* species were reported and are indicated in Table 1 [3,4,21].

Currently, not only humans suffer from the intoxication. Animals such as domestic pets, grazers and horses experience serious health effects. In Australia, for example, the setae produced by *Ochrogaster lunifer* (Table 2), from the family Notodontidae, cause equine amnionitis and fetal loss on horse farms. The abortions caused by these caterpillars cost horse owners approximately AUD 27–43 million every year [22].

In Europe, other Lepidoptera caterpillars sharing a similar setae-based defense system include *O. Lunifer,* causing plagues. In particular, wider public health implications in France and Italy are caused by the pine processionary caterpillar from the family Notodontidae (*Thaumetopoea pityocampa*) (Figure 1C, Table 2). In France, the incidence rates were 18%, and 60% from veterinary practitioners experiencing symptoms caused by the setae [22]. Other species of the genus *Thaumetopoea* also cause epidemic spreads of the airborne disease. In the summer of 2019, the oak processionary caterpillar (*Thaumetopoea processionea*) was responsible for a major impact on public health, with a soaring number of itchy dermatitis in Belgium, Netherlands, the United Kingdom, Germany and Austria (Figure 1D, Table 2) [3,23].

An important fact is that caterpillar envenoming is seen as an occupational disease. The specific populations at risk are chainsaw operators, people that need to climb in trees, and forestry and agricultural workers [22]. For example, in the rubber tree areas of Brazil, latex collectors are forced to stop their job because of a repeatable contact with *Premolis semirufa* of the family Erebidae (Table 1). This caterpillar is known to cause chronic symptoms similar to rheumatoid arthritis. Due to these devastating effects, the caterpillar was added in the “Manual of diagnosis and treatment of envenomation”, released by the Brazilian Ministry of Health [24].

In fact, everyone can be intoxicated, not only in the near vicinity of the caterpillar but also during outdoor activities and social events. As often as not, it seems that young children are more vulnerable because they play on the ground or in trees and the beautiful caterpillar colors attract curious children [25]. For some species, it is even known that the setae remain active for several years [26]. This means that at any time of the year, there is a risk for intoxication, thus posing a long-term threat to humans and animals [27].

Despite the various reported health problems, caterpillar envenomation remains an underestimated problem. The true number of accidents due to caterpillars worldwide is unknown. The World Health Organization does not report epidemiological data by all classes of venomous animals [28]. It is thus considered that the incidence rate of caterpillar envenomation is under-reported [14]. Moreover, there is a trend towards increased reporting of caterpillar envenomation cases. The rise in outbreaks is likely to continue and will be even of greater concern in the near future due to the triangle relationship between climate, international trade and local factors which can be explained further as follows. Global warming and international trade are greatly favoring the survival and distribution range of caterpillar species [23]. For instance, *T. processionea* has expanded its distribution northward. The main cause for this distribution is the observed warmer temperatures during winter and spring [23]. In Brazil, *L. obliqua* has traveled to neighboring countries, such as the province of Misiones in Argentina [14]. It is believed that this change in distribution is the result of human interventions to deforest the natural habitat. This forces the caterpillar to live in other regions with trees located in close proximity of people [15]. Hence, caterpillar–human contact is becoming more frequently reported. Also, local factors are playing a role in the increase in caterpillar envenomation. Regarding local effects, we refer to the extensive use of pesticides in recent years. This causes the death of many natural enemies of the caterpillar. Not only beneficial insects (wasps, stink bug and ants), but also valuable birds, such great tits, starlings, woodpeckers and *Anairetes alpinus* disappeared [29]. The worldwide incidence rates and the rise in the outbreaks emphasize the importance of recognizing caterpillar envenomation as an emerging health hazard.

## 3. Mechanism of Action

**Figure 2:**
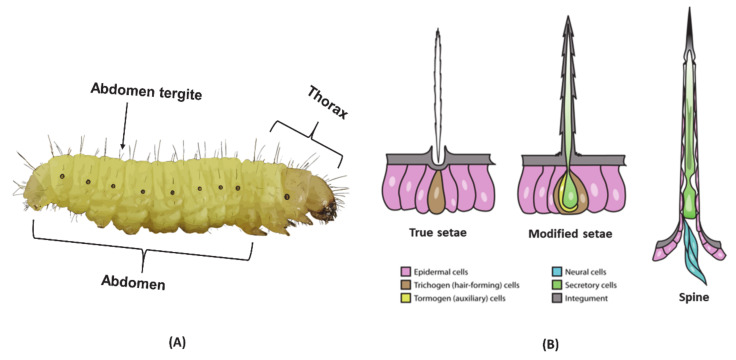
(**A**) Caterpillar morphology. (**B**) Schematic representation of setae/spine copied with permission from [44].

Caterpillars inject their venom through a specialized delivery system that includes harpoon-shaped setae or spines located all over the body and are more prominent on the integument of abdominal tergites, as shown in Figure 2A,B. As perfectly reviewed by Battisti et al. (2011) [44] and Villas-Boas et al. (2018) [4], the morphology, size and color vary depending on the family and species [4,44]. Different from other venomous insects, the larvae of a caterpillar lack a specialized venom gland [45]. Instead, venom is synthesized by secretory epithelial cells and stored inside the hollow canal of the setae [43]. The harpoon-shaped setae/spines have chitin-rich tips that easily break and thus serve as an injection needle for the venom. Once the venom is released from the hollow canal, the bioactive components can exert local and/or systemic pathological effects, acting on various organs in humans and animals.

## 4. Technologies to Analyze Caterpillar Venom Composition and Function

Classically, a gel-based strategy is applied to investigate the venom components inside the setae [46]. In this strategy, the complex mixture is first separated into different fractions by reversed-phase liquid-chromatography (HPLC). Venom components are then quantified with the results of sodium dodecyl sulfate polyacrylamide gel electrophoresis (SDS-PAGE). In this way, the researchers were able to visualize the complexity of the venomous mixture, but usually overlooked the low abundant components [47]. This limitation has sparked the development of refined methods to determine the venomous composition in more detail. Advances were provided by proteomics, transcriptomics and genomics. For proteomics, the gel-based strategy was combined with liquid chromatography tandem mass spectrometry (LC-MS) [22]. If the symptoms indicate an allergic reaction, an immune-proteome analysis may be performed to identify possible immunogenic components [48]. Immunoproteomics is a domain where immunoblotting is combined with mass spectrometry. Transcriptomic analysis (cDNA libraries, micro arrays and next generation sequencing) gives profiles of expressed genes in cells and tissues. It is a powerful tool to unravel the functional effects of toxins on cells and tissue. Above all, it also gives a better understanding of the pathways involved in the pathophysiology. For the genomic part, genomes are used to study gene function. However, big steps still need to be made for caterpillar species. For the majority of Lepidoptera superfamilies, there is no genomic database. So far, for six of the 43 Lepidoptera superfamilies (Bombycoidea, Geometroidea, Noctuoidea, Papilionoidea, Pyraloidea and Yponomeutoidea), at least one genome with a functional gene annotation is available [49]. For two superfamilies (Gracillarioidea and Hepialoidea), only a single genome without functional annotation exists [49].

**Figure 3:**
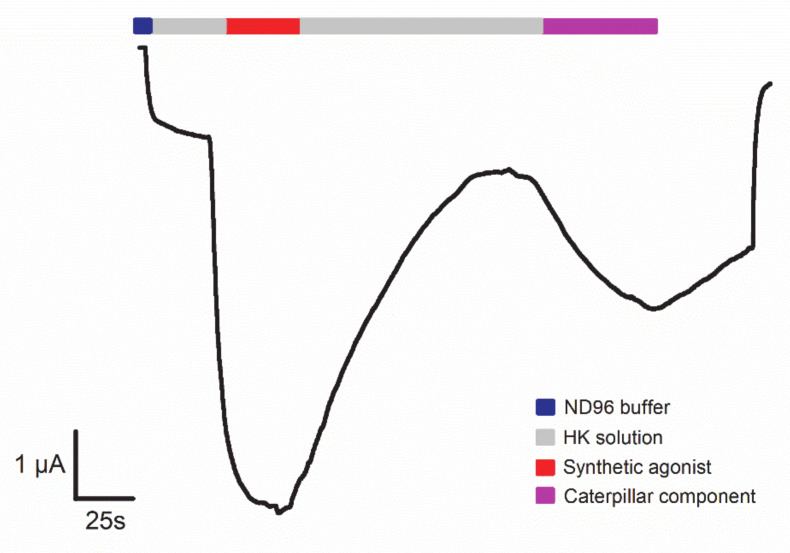
Electrophysiology-based bioassay with a two-electrode voltage clamp technique, as measured in *Xenopus laevis* oocytes. Shown is a validation trace experiment where a G-protein coupled receptor (GPCR) is coupled to an effector channel, an inward rectifier potassium channel via a G_i/o_ cascade. Currents were induced by exchanging a control saline low potassium solution (ND96 buffer = blue) with a measuring solution with elevated potassium (HK solution = grey). The trace reveals the agonistic activity of a bioactive component originating from a caterpillar (pink). A synthetic agonist was used as control.

All these analytical techniques are necessary for purification and preliminary characterization of the venom components. In addition to the characterization of the structure of the toxins, both primary and secondary, knowledge regarding the functional aspect is also required. A functional analysis could be investigated with a bioassay. An example of such a potent bioassay is an electrophysiological assay where the ligands are brought into contact with a molecular target in a heterological expression system. Figure 3 illustrates a typical example of such an electrophysiological bioassay in which a G-protein coupled receptor (GPCR) can be coupled to an effector channel. It enables researchers to investigate the bioactivity of venom components, for example from a caterpillar, in a specific and accurate way.

## 5. Venom Induced Pathophysiology and Diagnosis

**Figure 4:**
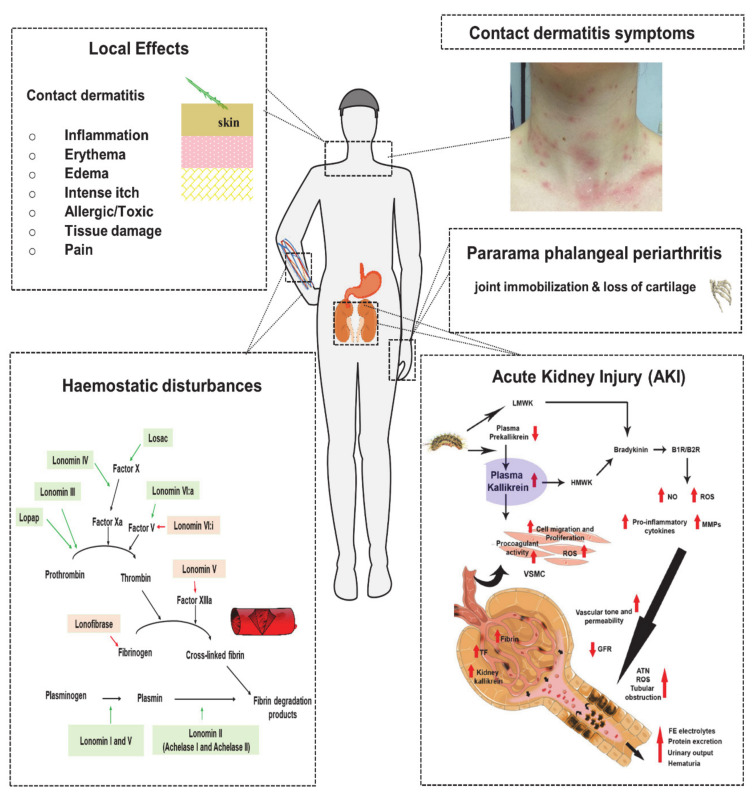
Venom components of different caterpillar species and their role on human body targets in the pathophysiology. Caterpillar venoms contain pharmacologically active components that are able to interfere with targets in the normal human cellular physiology. Some components are responsible for the local effects such as inflammation, erythema, edema, intense itch, tissue damage, pain and may exert an allergic reaction. Others affect the hemostasis by acting on the coagulation cascade or on the fibrinolytic pathway. Venom components colored in green activate a step in the cascade, while the components colored in red are able to inhibit a step. In some cases, this can lead to the development of an acute kidney injury (AKI). The venom-induced AKI cascade in the figure is copied with permission from [50].

In this part, identified bioactive components of different caterpillar species and their role on human body targets in pathophysiology are reviewed, summarized and illustrated in Figure 4.

### 5.1. Local Effects

Local effects are a hallmark physiological response to envenomation by many caterpillar species. Progress has been made in unraveling the underlying mechanism that causes the local effects. Different researchers identified venom components of several species and tried to link this information with the reactions seen in humans.

#### 5.1.1. Contact Dermatitis

##### 5.1.1.1. Allergic/Toxic Reaction

The most frequent clinical manifestation caused by caterpillar envenomation is the development of contact dermatitis, characterized by an extensive local inflammatory process with swelling, redness and an itch at the site of envenomation. This inflammatory environment is often initiated by an external trigger, a toxic compound, on a target in the human body. Therefore, several researchers looked at the venom composition of different caterpillar species and their target(s) to unravel the mechanism responsible for the provoked contact dermatitis symptoms.

One of the targets of caterpillar venom component(s) are mast cells. Mast cells are known to cause adverse skin reactions and play an important role in the pathogenesis of allergy, dermatitis, psoriasis and arthritis [51,52]. They are mainly disturbed in parts that are commonly exposed to the external environment, such as the skin, respiratory system and gastrointestinal tract [53]. Galicia-Curiel et al. (2014) [53] were the first to indicate that substances present in the setae of the Mexican caterpillar *Morpheis ehrenbergii,* from the family Nymphalidae, activate mast cells *in vitro* (Table 2). Once activated, mast cells release histamine which causes a rapid urticarial reaction. No massive degranulation was observed. Furthermore, the authors confirmed that no histamine was found in the crude venom extract. Moreover, no urticarial reaction was seen *in vivo* when the extract was preheated. This reinforces the hypothesis that a protein inside the setae is involved in an urticarial reaction since heat denatures proteins and does not affect histamine [53]. This is an interesting observation but certainly not a conclusive answer. Some targets are intrinsically temperature-dependent (e.g., TRPV1) and the binding of a ligand can alter the range of their temperature-dependence. Even in the absence of a ligand (setae extract), a change in temperature is sufficient in order to desensitize the targets and thereby stop the targets from responding [54].

Earlier, the same findings were also found in a study of the European caterpillar *T. pityocampa* of the family Notodontidae [55]. In this research, an abnormal permeability of surface blood vessels following the injection of venom was observed. This suggests an action of histamine, kinins and prostaglandins which are released after the activation of mast cells. Furthermore, dermal mast cell degranulation takes place 12 to 24 h after exposure to *T. pityocampa* venom extract. However, again, no excessive dermal mast cell degranulation was observed after contact with *T. pityocampa* setae. Thus, there remains the possibility that toxic substances inside the venom can either induce an inflammation process directly or indirectly via the activation of mast cells.

In the light of the research conducted by Lamy et al. (1983) [55], further investigations were performed on the venom content of *T. pityocampa* and other species in the family Notodontidae in order to gain more insight into the pathogenesis. The family Notodontidae is widely distributed in Europe (*T. processionea, T. pinivora, T. pityocampa and T. wilkinsoni*), Australia (*O. lunifer*), Africa (*H. semifusca*) and Asia (*Gazalina* sp.) [21]. Almost all species in this family are known as the leading cause of caterpillar epidemic airborne diseases, diagnosed with symptoms such as erythema, edema, papules, intense itch; swallowing can lead to a sore throat or difficulties with swallowing and contact with the eyes can lead to conjunctivitis [3]. The reaction may evolve to general malaise, fever or even an anaphylactic reaction [56]. This is mainly due to the ability to release urticaria setae as part of their protection mechanism. Such reactions were also seen in *Euproctis chrysorrhea* of the family Erebidae [57].

The European *T. pityocampa* is by far the most studied caterpillar in the family Notodontidae. Until now, three major proteins with allergenic activity have been identified and named thaumetopoein, Tha p 1 and Tha p 2. Thaumetopoein was the first specific protein fraction isolated from the setae of *T. pityocampa* with urticating properties. Electrophoretic techniques revealed two bands at 13 and 15 kDa [58]. Functionally, thaumetopoein has a direct non IgE-dependent effect on the mast cells [59,60,61]. However, an IgE-dependent effect via an immediate hypersensitivity reaction, responsible for more severe reactions (anaphylactic reactions), cannot be ruled out [27,59]. It is important to note is that Werno et al. (1993) [60] warned us to consider this protein as an important insect allergen. Unfortunately, thaumetopoein was never sequenced. As a consequence, researchers were not able to find homologies with other proteins or compare it. Tha p 1 is the second allergen found in the venom of *T. pityocampa,* with a molecular weight of 15 kDa and a N-terminal amino acid sequence GETYSDKYDTIDVNEVLQ [46]. This partial sequence does not show any homologies to other known proteins. Because of this, it is difficult to derive the biological function from the N-terminal amino acid sequence, which further increases the interest to study this protein in the future. Immunoblotting experiments could recognize the allergen in nine out of the 11 sera from patients sensitive to *T. pityocampa* [46]. Therefore, Tha p 1 is recognized as a major caterpillar allergen by the World Health Organization and International Union of Immunological Societies [62]. A third protein, unrelated to Tha p 1, with a molecular weight of 13 kDa and 115 amino acids, was successfully isolated and sequenced by Rodriguez-Mahillo et al. (2012) [48]. This third protein was called Tha p 2 [22]. In total, the researchers characterized 70 proteins, among them seven allergens, in the venom by using SDS-PAGE and immunoblotting techniques [48].

In 2015, Berardi et al. [40] added additional information about the structure of Tha p 2. They discovered that Tha p 2 is a glycine-, serine- and cysteine-rich protein that is present in the genome of all Notodontidae species investigated in the study (*T. bonjeani T. herculeana, T. ispartaensis, T. libanotic, T. pinivora, T. pityocampa, T. processionea, T. solitaria and T. wilkinsoni*) [22,40].

Cutting-edge technologies such as genomics, transcriptomics and proteomics made it possible to explore the venom of *T. pityocampa* in more detail [63]. By applying SDS-PAGE together with LC-MS/MS analysis, Berardi et al. (2017) [22] could identify 353 proteins in the complex mixture of *T. pityocampa*. The large amount of proteins found by LC-MS/MS compared to the study by Rodriguez-Mahillo et al. (2012) [48] is amazing. This shows even more how efficient and sensitive this technique is. Despite this, the characterization of Tha p 1 is still incomplete. The authors suggest that the isolation and full sequencing of the Tha p 1 could shed light on the function/nature of Tha p 1 [22]. In addition, serine proteases are found in the proteome with similarities to proteins in *Bombyx*, *Helicoverpa* and *Mamestra* [22]. Also, enzymes involved in chitin synthesis are noticed. It is known that chitin has a role in immune reactions. In any case, the role of a chitin-induced immune reaction due to contact with setae of caterpillars is not yet known. Furthermore, in clinical studies, it was noted that the majority of patients developed symptoms after 2-8 h. This suggests that the IgE-mediated immune pathway is relevant but does not explain in full the symptoms in patients [21]. Other mechanisms than those mediated by IgE must be at play. Some researchers, for example, refer to a toxic mechanism [64]. There is also no evidence for IgG antibodies participation [48]. Although a collection of diverse proteins has been identified in the venom of *T. pityocampa*, the targets of the venom component(s) are poorly understood. At this moment, there is no clear evidence of a correlation between an allergenic/toxic reaction and the already identified proteins. This information is necessary to unravel the mechanism of action.

In contrast with the amount of scientific literature on *T. pityocampa*, little is known about other processionary caterpillars. More research on the venom components of these species will undoubtedly shed light on the mechanism and may help to find a selective treatment options to adequately address the symptoms.

##### 5.1.1.2. Edema and Erythema

For *L. obliqua,* the greatest advancement has been made to understand the formation of edema and erythema. In the research conducted by De Castro Bastos et al. (2004) [35], the data revealed a significant inhibition of edema formation by the G protein-coupled histamine 1 receptor (H1R) antagonist, loratadine [35]. This suggests the involvement of histamine (released after activation of a target or due to a histamine-like component) in the formation of edema as response on the envenomation. The presence of histamine or histamine analogues was also found in other caterpillar species such as in *Dendrolimus pini* (family Lasiocampidae), *Euproctis chrysorrhoea* (family Erebidae), *Lymantria dispar* (gypsy moth, family Erebidae), *Doratifera oxleyi* (family Limacodidae), and *Latoia consocia* (family Limacodidae) [4]. Thanks to the contemporary knowledge we have of other isoforms (H2R, H3R and H4R), it is of course very interesting to gain scientific insight into whether the toxins or components of these caterpillar species can also interact with other histamine receptors and, if so, with what specificity.

On the other hand, it is also essential to note that for some caterpillar species, such as in *M. ehrenbergii* and *H. metabus*, no histamine was found in the venom [30,42,53]. In addition, many patients envenomed by setae of Lepidoptera species show a delayed onset of the dermal reactions. This suggests that rapid reactants such as histamine are not likely responsible for the effects observed [30,44]. Unfortunately, there is not much information about this mode of action yet.

Several years after the research by De Castro Bastos et al. (2004) [35], Bohrer et al. (2007) [65] added important information on the mechanism of the formation of edema. The researchers investigated the effects of the venom extract of *L. obliqua* on the kallikrein-kinin system (KKS). It was shown that the venom extract releases a kinin, kallidin, from low molecular weight kininogens (LMWK) and activates plasma pre-kallikrein. By the activation of plasma pre-kallikrein, the venom induces an indirect release of bradykinin from the high molecular weight kininogens (HMWK). Both bradykinin and kallidin are peptides that exert their biological effects by the activation of a G-protein coupled bradykinin receptor (B1/B2 receptor) [66]. In these experiments, it was observed that the rats developed edema and erythema in the peripheral tissues as a result of the activation of the B2 receptor (B2R). In this way, they could prove that the edematogenic action is not only mediated by histamine but also by the release of kinins. The same mechanism of KKS activation and resulting edema was also seen in other venomous animals, such as snakes, wasps and spiders [65].

Another study of the toxic content was performed on the female *H. metabus*. The female *H. metabus* from the family Saturniidae is mainly known in Venezuela, where a great number of envenomation accidents (Caripito itch) take place [31,32]. In the study conducted by Cabrera et al. (2015) [30], the researchers noticed that the edema generation by *H. metabus* was caused by the proteolytic activity of a fraction isolated by ammonium sulfate precipitation. In SDS-PAGE, two bands of 29 and 40 kDa were noticed [30]. Mass spectrometry revealed the presence of the same protein in the two bands. Moreover, a sequences alignment search of peptides yielded a significant homology with serine proteases of the S1A subfamily. The peptide, HM-PT60, was thus annotated as a S1 serine protease with a structural N-glycosylation by ESI-MS/MS. Next, the function of the N-glycosylated S1A serine protease was investigated in animal experiments. In these experiments, guinea pigs were inoculated with the toxin, which resulted in edema formation, massive fibrin deposition and hemorrhages. Also, an inflammatory process was activated since a leukocyte flux was observed. Histological analysis revealed that edema was formed as a result of the proteolytic activity of the toxin found in the venom. In fact, it seems that the optimal proteolytic activity of the toxin depends on the N-glycans present in the structure [30]. N-glycans are able to stabilize the 3D structure and/or optimize the enzyme-substrate binding by ionic interaction. In a subsequent research by Cabrera et al. (2017) [67], the authors could identify that the protein content of the setae is dominated by enzymes. More specifically, using SDS-PAGE analysis, they could identify that 65% of the venom content is represented by five proteases with homology to S1A serine proteases [67]. The function of these S1A serine proteases still needs more investigation, although for one of the proteases, HM-PT60, the function was already described as mentioned above [30]. Additionally, the data indicate the presence of a chitinase. Chitinases can break down chitins into small chitin fragments that may potentiate an inflammatory response. Moreover, the authors suggested that the combination of proteases and chitin can damage the exoskeleton of predatory arthropods. Slightly less important in causing the symptoms in humans, the caterpillar also contains vitellogenin, a bacteriostatic protein, which is important in the protection mechanism against pathogens. The observed tissue damage with hemorrhages induced by *H. metabus* will be further explained in the next topic about local tissue damage.

##### 5.1.1.3. Local Tissue Damage

Local tissue damage can appear at the site of envenomation as result of the action of some venom components. This favors the spread of toxins into the body which can lead to actions on the systemic level [36]. These actions are often provoked by the presence of hyaluronidases in the venom. Hyaluronidases are able to hydrolyze hyaluronan from the extracellular matrix which disturbs the extracellular matrix of tissue and blood vessel wall [37]. This disturbance facilitates the toxins to diffuse into the tissue. For that reason, these enzymes are known as toxin spreading factors for many venomous animals (snakes, wasps, scorpions, bees, spiders and caterpillars) [37]. For caterpillars, the presence of hyaluronidases has been reported in the venom of *L. obliqua*, *P. semirufa*, *M. urens* (Family Megalopygidae), *L. memusae*, and *Podalia* ca. *fuscescens* (Family Megalopygidae) [18,24,36,37]. For *L. obliqua,* the presence of hyaluronidases was proven by a zymogram experiment conducted by Gouveia et al. (2005) [36]. In this experiment, two hyaluronidases, lonoglyases, with a molecular mass of 49 and 53 kDa, were able to degrade hyaluronic acid. Specifically, they show an β-endohexosaminidase activity which enables them to produce terminal N-acetylglucosamine sugar residues after cleaving hyaluronic acid. In addition to hyaluronic acid, they can also cleave chondroitin sulfate residues linked to the extracellular matrix (ECM). The most optimal activity was observed within the pH range 6–7 [36]. No activity was detected below pH 5 and above pH 8. This means that the hyaluronidases are active under normal physiological conditions. All these observations may explain the disturbances of cell adhesion and migration events that are seen after envenomation [36]. Moreover, the authors speculate that there is a synergy with other toxins in the venom, a phenomena also seen in other venomous animals [68]. This could explain the formation of local hemorrhaging, characterized by a disruption of the subendothelial extracellular matrix causing blood vessel wall instability [36]. It is important to emphasize that the activity of hyaluronidases of many caterpillar venoms may lie at the root of the development of local tissue damage that evolves to a systemic effect.

In contrast, very low activity towards hyaluronic acid was found in the venom from *Megalopyge lanata* and *Podalia orsilochus* (Table 2) [41]. In *Lagoa crispate,* no hyaluronidases were found (Table 2) [42]. The listed caterpillars belong to the family of Megalopygidae. The first two are widely distributed in the Neotropics, whereas *L. crispate* accidents are mainly reported in Oklahoma. Envenomation by these caterpillar species is mainly characterized by the appearance of cutaneous reactions such as pain, edema and erythema. Systemic troubles are not really attributed to these caterpillars.

Furthermore, Berger et al. (2013) [69] confirmed the occurrence of myotoxins in the venom of *L. obliqua*. Using *in vivo* experiments, the authors saw an increase in creatine kinase and histologically muscle damage in subcutaneous tissue (at the contact site) and myocardial necrosis [69]. For *L. obliqua*, the toxins responsible for this myotoxic activity are still unknown. Contrarily, for many snake venoms, these myotoxins have been identified. Here, phospholipase A_2_ enzymes are accountable for the damage to the muscles and the development of necrosis [70]. Phospholipases A were also found in the venom of *E. chrysorrhoea* and *E. subflava* caterpillars and are presumably responsible for the cutaneous reactions seen in patients envenomed by these species.

It is important to highlight is that the venom-induced local tissue injury caused by *P. orsilochus* is quite similar to the one observed after *L. obliqua* envenomation. In the research of Sánchez et al. (2019) [41], they noticed the presence of serine proteases in the venom by applying a gel electrophoresis technique. This type of enzyme could perhaps explain the observation that the venom is able to hydrolyze both fibrinogen and fibrin with a specificity towards the α chain of both molecules and which may lead to the observed vascular lesion [41]. Furthermore, skin microscopy revealed not only the presence of vascular lesions, but also an inflammation reaction, hemorrhage and necrosis [41]. Some researchers proposed that the cell necrosis in the epidermis results from the reaction with lepidopteran toxin(s). Furthermore, no muscle necrosis could be determined. This perhaps can be explained by the fact that no phospholipase A_2_ enzyme activity was detected. In addition, hemorrhaging started several hours after the start of necrosis. The toxin(s) or molecules that are responsible for the development of necrosis are still under investigation but the scientists suggest a mechanism where the toxin(s) or components act directly on the cell membrane [41].

Tissue damage, such as erosion of capillary vessels and focal hemorrhage, was also observed after envenomation by *H. metabus* [30]. The action is most likely caused by the proteolytic activity of S1A serine proteases found in the venom, as described previously. Furthermore, an inflammatory process was observed. The activation of the innate immune system was mediated by the structural presence of sulfate groups linked to the N-glycans [30]. Above all, it is believed that antigens are recognized, which results in the influx of leukocytes, fibrin deposition and activation of blood coagulation cascade by neutrophils.

##### 5.1.1.4. Pain

When a venomous extract of setae/spines comes into contact with human skin, it can induce an intense (burning) pain sensation (Table 1 and Table 2). In more severe cases, patients describe this pain as similar to having “a hot coal applied to the skin” or “being hit on the arm with a baseball bat” [71]. A pain signal, in general, is generated by increasing the sensitivity of nociceptor. This activation results in the release of chemical factors from nociceptors or non-neural cells (e.g., mast cells, basophils, platelets, macrophages, neutrophils, endothelial cells, keratinocytes and fibroblast) [72,73]. The chemical factors such as serotonin, histamine, glutamate, ATP, adenosine, bradykinin, prostaglandins, interleukin 1β (IL-1β), extracellular proteases and tumor necrosis factor α (TNF-α) interact directly with the nociceptor by binding with one or more cell surface receptors (G-protein coupled receptor, transient receptor potential (TRP), acid-sensitive ion channels (ASIC), voltage-gated ion channels (VGICs), two-pore potassium channels (K2P), and receptor tyrosine kinases (RTK)) [72,73]. This information is necessary to understand and to link the role of some venom components with the pathogenesis of pain.

For *L. obliqua*, de Castro Bastos et al. (2004) [35] studied the nociceptive responses elicited by a crude extract *in vivo* in rats. Pain was assessed by investigating the change in shaking and lifting of the injected hind paw. Surprisingly, they saw that the shaking and lifting was strongly inhibited after treatment with indomethacin. Indomethacin is a pain medicine that works by inhibiting the production of prostaglandins [74]. The nociceptive responses were thus elicited by prostaglandins. Therefore, the authors proposed that prostaglandins were formed by the phospholipase A_2_ activity found in the spine extract of *L. obliqua* [8,35]. Phospholipase A_2_ is a low molecular mass enzyme (15 kDa) that produces arachidonic acids which can be converted into prostaglandin by cyclooxygenase [75]. Other researchers suggested that the KKS mechanism, which was also involved in the formation of edema, may play an important role in the pain response that humans experience after envenomation. The KKS mechanism releases bradykinin, which is a powerful pain mediator. It namely sensitizes nociceptors following the release of prostaglandins and cytokines [65].

**Figure 5:**
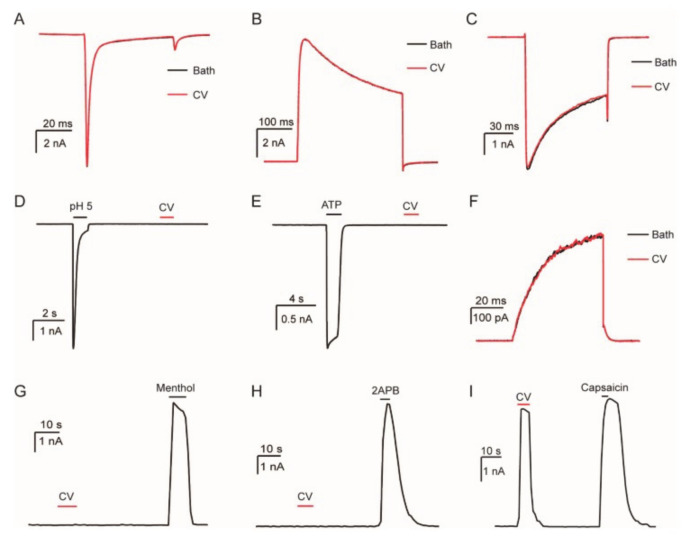
In these experiments, Yao et al. (2019) [43] tested the response of the crude *L. consocia* venom (CV) extract on pain-related ion channels: (**A**) dorsal root ganglion sodium channel (DRG-Na); (**B**) dorsal root ganglion potassium channel (DRG-K); (**C**) dorsal root ganglion calcium channel (DRG-Ca); (**D**) acid-sensing ion channel 2a (mASIC2a); (**E**) P2X ligand-gated ion channel 3 (hP2X3); (**F**) mKCNQ4; (**G**) transient receptor-potential M8 (mTRPM8); (**H**) transient receptor-potential vanilloid 2 (mTRPV2) (**I**) TRPV1. The electrophysiological profiles of *L. consocia* venom reveals a potent and specificity towards the TRPV1 channel with similar amplitude to the agonist, capsaicin, evoked current (**I**). No evoked currents were seen for the other channels (**A–H**). The figure was copied with permission from [43].

It seems that not only the KKS mechanism may be involved. Both genetic and electrophysiological studies with whole-cell patch clamp have, in fact, shown that transient receptor potential vanilloid 1 channel (TRPV1) is one of the primary targets of the *L. consocia* venom (Figure 5) [43].

**Figure 6:**
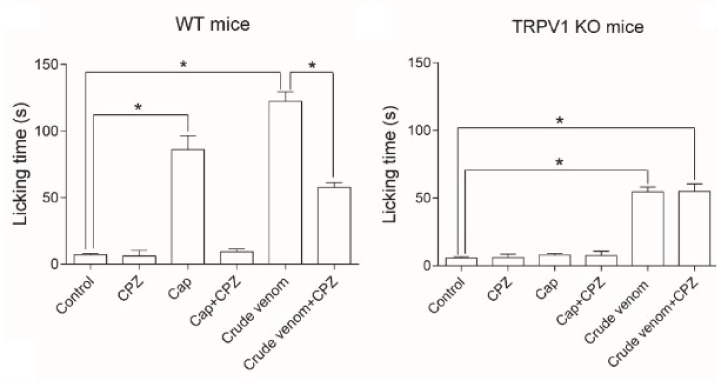
The TRPV1 antagonist, capsazepine, could not completely eliminate the paw licking behavior. Paw licking duration was monitored by Yao et al. (2019) [43] using the following experimental conditions. (A) Ten microliters of saline (control), capsazepine (CPZ, 2 mM), capsaicin (Cap, 500 µM), crude venom (100 µg/mL), capsaicin (500 µM)/capsazepine (2 mM) mixture, and crude venom (100 µg/mL)/capsazepine (2 mM) mixture injected into the left hind paw of WT mice. Two-sided *t*-test: *, *p* < 0.05; n = 6. (B) Mean durations of paw licking induced by 10 µL of saline (control), capsazepine (2 mM), capsaicin (500 µM), crude venom (100 µg/mL), capsaicin (500 µM)/capsazepine (2 mM) mixture, and crude venom (100 µg/mL)/capsazepine (2 mM) mixture injected into the paw of TRPV1 KO mice. The figure was copied with permission from [43].

*L. consocia* is a caterpillar found in South-West China that induces an intense burning pain sensation. By targeting the sensory nerve endings of the TRPV1 channel, it mediates nociceptive triggers from the peripheral nervous system to the central nervous system where it is received as pain. Additionally, Yao et al. (2019) [43] did not rule out that more pain-related receptors are involved in the pathway. In their *in vivo* experiments, they saw that the paw licking behavior of the mice was not completely eliminated by a specific TRPV1 antagonist, capsazepine (Figure 6). According to the observations, they mainly believe that there is a possibility that other targets work synergistically with the TRPV1 channel to induce pain. Additionally, the authors investigated the venom composition of the caterpillar. They were able to identify 126,670 universal genes, 162 of which are related to the defense mechanism. Overall, they could demonstrate that the venom is rich in peptide fragments. Although more research still needs to be performed to indicate the bioactive components and their target in the pain pathway of *L. consocia*, it appears that venomous animals that induce pain usually have venom components acting on TRP channels. For example, the DkTx toxin of the Chinese bird spider, *Ornithoctonus huwena*, activates TRPV1 channels and causes severe pain [76]. Also, the Chinese red-headed centipede (*Scolopendra subspinipes*) and some scorpion venoms induce pain by the same pathway [43].

### 5.2. Systemic Effect

Just as beauty is not inherent in a visual image, envenomation by caterpillars is a complex clinical manifestation that often not only involves local effects but also systemic effects. Frequently, the clinical profile after envenomation by some caterpillar species, e.g., *Lonomia* species, begins with burning pain, dermatitis, headache and nausea symptoms but evolves rapidly into a hemorrhagic syndrome accompanied by acute kidney injury (AKI) [10].

#### 5.2.1. Haemostatic Disturbances

Hemostatic disturbances are a major consequence of envenomation by some caterpillar species and, more specifically, by *Lonomia* species. They not only occur at the site of the accident but may manifest at the systemic level and consequently lead to abdominal or brain hemorrhage. Also, consumptive coagulopathy can occur [36]. For two *Lonomia* species, *L. obliqua* and *L. achelous*, some responsible mechanisms are well described, but still a lot of information remains not entirely elucidated. Although the pathophysiologic processes are not completely known, it seems that depending on the species, another mechanism is involved in this hemostatic disturbance. The effect of some toxins from *L. obliqua* and *L. achelous* are highlighted in Figure 4 and further explored below.

For *L. obliqua*, important information was derived from the proteome and transcriptome analysis. Researchers found sequences from 1278 independent clones assembled into 702 clusters of genes coding for putative toxins such as lipocalins, blood coagulation factors, phospholipase A_2_ hemolins, cystein-proteases, serine-protease inhibitors, serpins and hyaluronidases [5]. Firstly, the most abundantly studied toxin was found in the lipocalin protein family, called Lopap (*Lonomia obliqua* prothrombin activator protease) [77]. Lopap is a 69 kDa toxin and the first lipocalin described with proteolytic activity. Hence, it activates prothrombin through hydrolysis of Arg^284^-Thr^285^and Arg^320^-Ile^321^ peptide bonds, which leads to the generation of thrombin [8]. It is surprising that Lopap did not show any similarity with other prothrombin activators or serine proteases but only with lipocalins. As a result of the thrombin formation, Lopap may indirectly induce a platelet aggregation. In contrast, Lopap could also inhibit platelet aggregation [77]. This inhibition results from the release of cell platelet aggregation inhibitors from endothelial cells such as nitric oxide (NO) and prostacyclin (PGI_2_). Besides the inhibition of platelet activation, NO and PGI_2_ display potent vasodilation properties [5]. All these factors play an important role in the venom-induced consumption coagulopathy, extended defibrinogenation and incoagulability [78]. Clinically, it will result in an alteration of blood clotting tests after the *in vivo* administration of Lopap. The resulting condition may evolve into systemic bleeding, especially when the integrity of blood vessels is disrupted [33]. Secondly, a 35 kDa enzyme (Lonofibrase) isolated from the hemolymph with fibrin(ogen)olytic activity was found. Lonofibrase cleaves Aα and Bβ chains of fibrinogen. The specificity of Lonofibrase against the α- and β-chains of fibrin is sufficient to prevent the clotting reaction, which explains the severe hemorrhagic clinical profile seen. Next, a 45 kDa Factor X activator, known as Losac (*Lonomia obliqua* stuart factor activator), was found. When Factor X is activated, it will integrate the prothrombinase complex (phospholipids and calcium ions) to produce thrombin. Consequently, a fibrin clot is formed [79]. For many snake venoms, it is known that they contain enzymes that activate Factor X [80]. The only difference with Losac from *L. obliqua* is that these enzymes critically depend on the presence of calcium ions. Losac is able to activate Factor X in the absence of calcium ions. It seems clear that proteolytic activation of Factor X is made possible due to the serine like protease activity of Losac since its action can be abolished by a serine protease inhibitor, diisopropyl fluorosphosphate[8]. Interestingly, Losac does not show homology to known proteases, but instead it has a high similarity with the hemolin group. Hemolin is an immunoglobulin-like peptide with an important role in insect immunity and cell adhesion properties [79]. It is exclusively expressed in Lepidoptera species. Moreover, experimental thrombosis studies showed that Losac has no effect on fibrin or fibrinogen [79]. This again reinforces the evidence that Losac has an important and specific roll in inducing blood coagulation. Beyond the role in coagulation, Losac is also able to induce proliferation and inhibit apoptosis [79,80]. Moreover, the occurrence of intravascular hemolysis was confirmed by *in vivo* experiments in rats [8]. This hemolytic process is most likely initiated by the deposition of hemoglobin in the renal tubes which is highly nephrotoxic [69]. One important contributor to the hemolytic activity is a 15 kDA group III phospholipase A_2_ found in the venom of *L. obliqua*. Furthermore, proteomic data of *L. obliqua* venom demonstrated the presence of elements that modulate cell adhesion and signaling. This is probably mediated by the activation of RAC1 signaling pathway. RAC1 is a member of the Rho GTPase family and promotes actin assembly, which influences the cell function due to changes in cell adhesion and migration [81]. Finally, physicians often note a sudden drop in blood pressure in the patient. Bohrer et al. (2007) [65] revealed that this hypotension was mediated by the activation of the KKS system. This was proven by the inhibition of hypotension by aprotinin, a plasma kallikrein inhibitor, and HOE-14, a bradykinin B2 antagonist [35].

While *L. obliqua* venom predominantly acts on the coagulation cascade, *L. achelous* mainly affects fibrinolysis [8,33]. Several studies of *L. achelous* hemolyph supported the hypotheses that different venom fractions play a role in haemostatic disturbance, including Lonomin I, Lonomin II (Achelase I and Achelase II), Lonomin III, Lonomin IV, Lonomin V, Lonomin VI:a, Lonomin VI:I, and Lonomin VII [8,82]. The actions of these venom components are visualized in Figure 4 and further explained below.

Two venom fractions, Lonomin I and Lonomin II (Achelase I and Achelase II), have an intense fibrinolytic activity and can induce lysis of whole blood clots and fibrin plates. Lonomin I is a 16 kDa venom toxin with urokinase plasminogen activator-like activity and Achelase I (22.4 kDa) and Achelase II (22.7 kDa) have a plasmin-like activity. Furthermore, two prothrombin activators are found. Lonomin III can activate prothrombin independently from calcium ions and phospholipids (prothrombinase complex). Meanwhile, Lonomin IV is a factor Xa activator, since its activity increases in the presence of factor V, phospholipids and calcium ions. Next, *L. achelous* venom contains a Factor V activator, Lonomin VI:a, and inhibitors, including Lonomin VI:I [83]. Lonomin VI:a appears to be a thermostable metalloprotease, since its activity is inhibited by metalloprotease inhibitors. For a variety of snake venoms, it is known that they have Factor V activators in their venom, mainly responsible for the progression of hemorrhaging. In contrast, the venom also contains a Factor V inhibitor, Lonomin VI:i, which is a serine or cysteine protease. Besides Lonomin II, Lonomin V is the second most important enzyme, with intense fibrinolytic activity. Lonomin V is a 25 kDa factor XIII proteolytic urokinase-like enzyme that degrades the extracellular matrix proteins such as laminin, vitronectin and fibronectin [8]. The adhesion and aggregation inhibition in platelets destroy the capillaries and contributes to the hemorrhagic syndrome. Additionally, its activity is inhibited by serine protease inhibitors, suggesting that it is a serine protease. At last, thrombosis experiments on the whole venom indicate that Lonomin V not only inhibits thrombus growth but also induces lysis of preformed thrombi [8].

**Figure 7:**
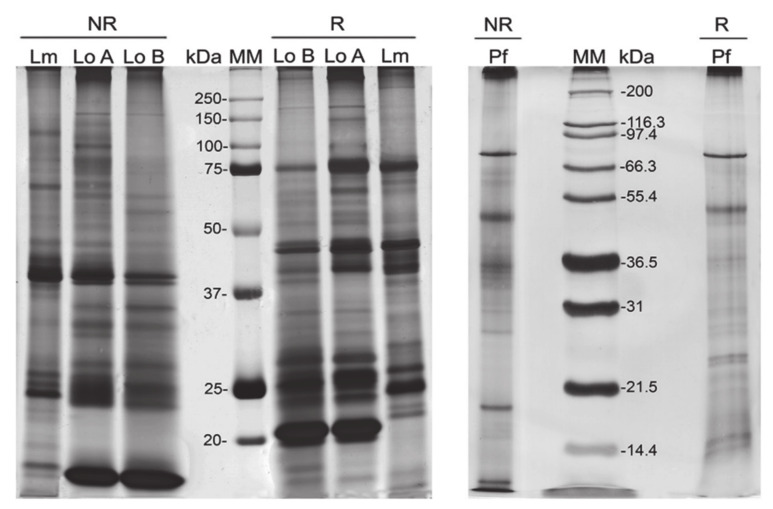
One-dimensional electrophoretic profile of (**A**) Saturniidae and (**B**) Megalopygidae venom extract under reducing (R) and non-reducing (NR) conditions. Lo A: *Lonomia obliqua* from Argentina; Lo B: *Lonomia obliqua* from Brazil; Lm: *Leucanella memusae*; Pf: *Podalia* ca. *fuscescens*; MM: molecular mass markers. The figure was copied from Quintana et al. (2017) [18] with permission from Elsevier.

Besides their presence in *Lonomia* species, proteins related to the haemostatic disturbance were also identified in other species from the same or different family. Quintana et al. (2017) [18] investigated the protein content of *L. obliqua*, *L. memusae* and *P.* ca. *fuscescens* and compared it. In a one-dimensional electrophoretic profile, shown in Figure 7, it is clearly visible that the venom of Saturniidae species (*L. obliqua* and *L. memusae*) are more complex compared to the venom of *P.* ca. *fuscescens* from the Megalopygidae family. Notably, the same coagulation disturbance proteins such as serpins and serine proteases from *L. obliqua* were also found in *L. memusae*. In contrast with the intense presence of Achelase 2 (anticoagulant protein) and serine proteases in *L. obliqua* venom, lower intense bands were noticed in *L. memusae* venom. Clinically, the patients showed a prolonged activated partial thromboplastin time of the normal human plasma (APTT). This is a blood test that characterizes the coagulation of blood. The authors suggest that the action is caused by the proteolysis of coagulation factor(s). However, the venom did not degrade human fibrinogen [18].

Another important result of the research conducted by Quintana et al. (2017) [18] is that Losac, a hemolin of *L. obliqua*, was detected in the venom of *L. memusae*. Surprisingly, the same component, Losac, is also found in other species of the Megalopygidae family such as *Podalia* ca. *fuscescens* and *P. orsilochus* (Table 2). Just like *L. obliqua*, both species are able to hydrolyze fibrinogen and fibrin with a particular preference towards the α chain of both fibrinogen and fibrin (as described in the section Contact Dermatitis with local tissue damage for *P. orsilochus*). Indeed, envenomated patients show a shortening of the clotting time of human plasma, similar to *L. obliqua* envenomed patients [18,41]. Of note, *P.* ca. *fuscescens* needs more time to act on the plasma protein. This action may be due to a weak coagulant component in the venom. Serine proteases and serpins are also found in both species. The only strange observation which is important to emphasize is that for *L. memusae*, *P. orsilochus* and *P.* ca. *fuscescens*, no clinical symptoms of hemostatic disturbance have been reported yet [41]. Overall, it was hereby again shown that enzymes are one of the most common toxic components found in caterpillar species [67,84].

#### 5.2.2. Acute Kidney Injury

The development of an acute kidney injury (AKI) is associated with a high morbidity and mortality. Envenomed patients experience a sudden loss of basic renal function, such as filtration and excretion capacities and the maintenance of homeostasis [15,50,85]. Berger et al. (2019) [50] were the first to expose the mechanisms associated with the pathogenesis of renal damage by *L. obliqua*. The results are summarized in Figure 4.

Firstly, they discovered that bradykinin, released from LMWK (directly) and HMWK (indirectly), as described previously in the section Contact Dermatitis with Edema and Erythema, contributes to kidney injury mainly by the activation of its two receptors, B1R and B2R [50]. Both receptors belong to the G-protein-coupled receptor group. It is important to note that under normal physiological conditions, B1R is not expressed, but may be upregulated by an inflammatory stimulus [66,85]. When B2R is stimulated with bradykinin, a vasorelaxant effect was observed, while the activation of B1R by the bradykinin metabolite, Arg^9^-bradykinin, resulted in a renal vasoconstrictor response. The renal vasoconstrictor response induces a decrease in glomerular filtration rate and electrolyte balance. Additionally, it was observed that *L. obliqua* venom induces the production of several cytokines, increased the expression of matrix metalloproteinases (MMPs) and increased the levels of nitric oxide (NO). As a consequence, these alterations resulted in tubular lesions and hereby the role of the activation of kininogen-kallikrein-BK-B1R/B2R in *L. obliqua* envenomation was shown [50]. Moreover, this work pointed out that cytokines and coagulation factors are produced in the plasma during the envenomation. Also, activation of vascular smooth muscle cell (VSMC) procoagulant activity, increased reactive oxygen species production (ROS), and cell proliferation and migration were observed [50]. The venom can also act directly on the VSCMC and thus cause the same cascade of events as plasma [6]. At last, researchers have asserted that renal obstruction or reduced filtration capacity is caused by activation of the VSMC by the venom components that lead to fibrin formation and deposition in glomerular capillaries. All these findings and their role in the pathogenesis of AKI are summarized and presented in Figure 4. Other species of the Saturniidae family, such as *Dirphia* species and *L. achelous,* are also known to induce AKI [34].

#### 5.2.3. Pararama-Associated Phalangeal Periarthritis

Pararama-associated phalangeal periarthritis is an occupational and serious health disease in the Brazilian Amazon region. The name of disease is derived from the caterpillar causing the disease, i.e., *P. semirufa* or pararama. *Premolis* species belongs to the Erebidae family and are found in South America, where they feed on the *Heyea brasiliensis*, the rubber trees (*P. semirufa* in Brazil, French Guiana, Ecuador, Peru and Panama; *P. excavata* in Panama; *P. rhyssa* in Peru and *P. amaryllis* in French Guiana) [24]. The disease is a unique form of erucism that begins with symptoms such as pain, itch, heat and redness but may rapidly escalate to pararamose or joint immobilization, loss of cartilage and bone structure when repeated contact with the setae of the caterpillar occurs (Figure 4) [38]. In fact, one can compare this chronic situation with the symptoms caused by an inflammation joint disease such as rheumatoid arthritis. The envenomation by *Premolis* species is not only seen as an occupational disease, but it can also have a serious social impact [39].

Villas-Boas et al. (2012) [24] took the first steps to investigate the venom composition of *P. semirufa* in order to gain more insight into the mechanism. Soon it became clear that the venom has a complex composition with different enzymes acting alone or together to generate and develop the clinical symptoms. Firstly, they found hyaluronidases in the venom. As described in the section Contact Dermatitis with Local Tissue Damage, these enzymes degrade the extracellular matrix of the tissue, which facilitates the systemic influx of toxins. The link with the loss of cartilage and joint immobility can be found in the important function of hyaluronic acid. Hyaluronic acids are essential substances of the intercellular matrix of the skin, cartilage and synovial fluid that stabilize the joints and acts as lubricant [24]. A degradation of these hyaluronic acids results in the pararama-induced loss of cartilage and bone structure. Thus, the authors suggested that hyaluronidases are important factors behind pararama-associated phalangeal periarthritis.

Secondly, a serine protease with gelatinase activity was found. The gelatinase activity is often related with degradation of type IV, V, VII and XI collagens that are present in bone and cartilage. This protein is also related with a serine protease activity [24]. As described in a previous section about hemostatic disturbance, these serine proteases have diverse pharmacological activities. Some of them work on coagulation cascade by activating Factor V, whereas others have a fibrinogenolysis activity and activate plasminogen [24]. As a result, these compounds may be involved in the clinical manifestation. In contrast with many other venomous animals and other caterpillars, *P. semirufa* venom extract does not exert a phospholipase A_2_ activity.

Also, the participation of the immune system in the pathogenesis of pararama was investigated by Villas-Boas et al. (2013) [34]. Immunohistochemical and immunofluorescence research on BALB/c mouse injected with the venom revealed an elevated level of neutrophils in the connective tissue at the site of injection [34]. Besides their role in the innate immune system, neutrophils can also promote tissue injury and may be responsible for the inflammatory responses. In addition, the obtained results indicate an influx of macrophages which produce cytokines such as TNF-α, IL-1β and IL-6. In this way the inflammatory response is maintained. Moreover, the level of other cytokines, IL-6, IL-12, IL-10, IL-17 and IL-23 was increased [34]. Also, proliferation and activation of T and B lymphocytes was observed. The exact function and response of T and B lymphocytes on the venom remains to be elucidated. In a subsequent study in 2015, the same author added important information to the function of the serine proteases. According to the data, a serine protease containing fraction (Ps82) is able to activate the complement system and release anaphylatoxins [39]. This excessive complement system activation starts the development of an inflammation process. The same action is also seen in other venomous animals. For example, in many snake venoms, toxins were described to target the complementary system causing the observed pathogenesis [86].

## 6. Clinical Time Course

The symptoms that occur after envenomation by caterpillars can be divided into immediate, short- and long-term effects.

### 6.1. Immediate Effects

The immediate emerging effects appear within minutes after exposure to the setae/spines. These effects may vary depending on the type of species and the person envenomed. In many cases, a feeling of pain is experienced, which is obvious since caterpillars use their setae/spines purely as defense. In a specific case of *L. obliqua*, the victim experienced a sharp pain immediately after contact with the caterpillar [10]. Other species, more specifically, processionary caterpillars, are known to cause a direct intense itch reaction. Sometimes, patients are sensitized because of a repeatable contact with the caterpillar. In such case, the clinical pattern progressively evolves and may result in a severe life-threatening form of a systemic allergic reaction, as seen in *T. pityocampa* and *T. processionea* envenomation [26,61].

### 6.2. Short-Term Effects

Short-term effects are mainly the skin reactions seen at the place of the envenomation. These skin reactions are characterized by the formation of papules, pustules, swelling, acute inflammation and erythema and appear usually within 4–12 h (a delayed onset). In more rare cases, tissue necrosis in the area of contact may progress. The complete disappearance of these symptoms and restoration of the normal function takes 1–2 weeks, but may persist for up to a month [87,88]. In some cases, the setae of caterpillars are spread in the air by the wind. This can cause the setae to be inhaled. Usually, the victim will then develop a sore throat and experience difficulties with swallowing within four hours. Sometimes breathing problems, vomiting or abdominal pain may develop. Although vomiting or abdominal pain is not always related with inhalation of the setae, it is possible that these symptoms indicate a systemic envenoming resulting from the action of some specific component(s) inside the venom.

### 6.3. Long-Term Effects

In some caterpillars, the effect of the toxins may lead to a permanent loss of function. For example, when setae of processionary caterpillars penetrate into the mucous membrane of the eye, inflammatory signs (ophthalmia nodosa) can progress within 1–4 h [89]. If these setae are not removed, it can lead to irreversible blindness. Another example is related to the pararama caterpillar in South America. After repeated contact with this caterpillar, the patient may develop chronic symptoms similar to rheumatoid arthritis (as described in the section Venom-Induced Pathophysiology) [24]. For *L. obliqua*, the clinical profile may become even worse. Systemic effects begin with nausea and headache symptoms that become obvious after 24 h and can last about 72 h. Hemorrhagic vesicles are usually noticed four days after envenomation. Usually, blood tests show a prothrombin activity lower than 10% and undetectable plasma fibrinogen [10]. Abdominal pain, bleeding and coagulopathy also develop and may persist when the patient is not treated. If on day seven hematuria persists and the symptoms remain untreated, the patient will likely die [10]. Therefore, it is important to respond quickly and deliver the antivenom to save the patient.

## 7. Treatments and Limitations

Caterpillar envenomation has become a serious health problem in the world, but especially in the tropical zones, where it even can lead to fatalities. Prevention, proper management and an effective treatment option play an important role in minimizing discomfort and treat the complication after caterpillar envenomation. However, the presence of a treatment to adequately address the conditions of the envenomation by many caterpillar species is still lacking. Of course, it is obvious that the best preventive measure is to avoid any direct or indirect contact with caterpillar species. However, the real question everyone wants to get answered is what to do if one is intoxicated with the setae of a caterpillar. At this moment, doctors have limited means at their disposal and mainly propose supportive treatments to relieve symptoms provoked by many caterpillar species.

### 7.1. Antivenom for Management of Systemic Effects

In response to an outbreak in 1989 of *L. obliqua* species with a great number of accidents and mortalities, the Brazil institution Butantan in São Paulo developed a *Lonomia* antivenom to treat victims of *L. obliqua* envenomation [90]. For many venomous animals, an antivenom treatment is the standard-of-care to treat envenomation. However, for caterpillar species, this was the first and only antivenom manufactured in the world. The antivenom was made by immunizing horses with the venom extract from the spines of *L. obliqua* over a period of one year [90,91]. As a reaction, horses produced antibodies that were able to recognize and neutralize the coagulopathy effect of the toxic substances inside the venom. From the plasma of the horses, researchers obtained the specific purified F(ab’)2 immunoglobulin which forms the basis of the antivenom. This antivenom was manufactured to treat envenomed patients and showed an ED_50_ and potency of 38.61 µl and 0.29 mg/mL, respectively [91]. It is important to note that the introduction of this antivenom on the market in 1994 in Brazil significantly reduced the mortality rate, but unfortunately it could not be reduced to zero. People continue to die after envenomation by *L. obliqua*. As mentioned in the epidemiology section, this is mainly due to the development of an acute kidney injury [15]. The reason why this happens was exposed in the research by Berger et al. (2019) [50] and described in the section Venom-Induced Pathophysiology. By an experimental model of induced AKI, the researchers could highlight that a pharmacological inhibition of kallikrein with aprotinin results in restoring the renal function. Therefore, they suggested that a kallikrein inhibitor can be used as a promising therapeutic option complementary to the antivenom therapy, thereby reducing the mortality rate.

As noticed, the *Lonomia* antivenom is made based on a *L. obliqua* venom extract. Therefore, researchers want to investigate the efficacy and possibility of using the antivenom to treat envenomation by other *Lonomia* species. Sano-Martins et al. (2018) [92] were the first to investigate this and indicated that the *Lonomia* antivenom can restore the hemostatic disturbances caused by contact with *L. orientoandensis* and *L. casanarensis* in Colombia [92]. In Venezuela, the recommended treatment of *L. achelous* is mainly based on the use of antifibrinolytic medicines or replacement therapy of the whole blood, plasma or cryoprecipitates. This is in contrast with envenomation by *L. obliqua.* Gonçalves et al. (2007) [93] observed a high death rate in rats that were envenomed with *L. obliqua* and treated with an antifibrinolytic drugs, such as epsilonaminocaproic acid (EACA). The data indicate that the use of EACA could worsen the coagulation disturbances and that the *Lonomia* antivenom is probably the only effective treatment option today [94]. The differences in pathophysiology induced by both species may explain this observation.

*Lonomia* antivenom is only manufactured by the Instituto Butantan in Brazil. Yearly, several vials of the antivenom are distributed to the whole country by the Ministry of Health in Brazil. This means that according to the law, there is no authorization to commercialize the *Lonomia* antivenom outside Brazil. Due to this, physicians must request a Temporary Use Authorization delivered by the National Agency of Drugs to use the *Lonomia* antivenom outside Brazil. An example of such a case occurred in 2018 [10]. In this case, a good cooperation between all parties ensured that a patient in French Guiana could be saved and recovered without complications.

Despite the life-saving effect of the *Lonomia* antivenom, it also has some therapeutic limitations [28,50]. (i) Conventional antivenom vials contain around 70% or more IgG that are not specific against the venom proteins. These are IgGs that the animal has produced against antigens encountered during its lifetime. These extra antibodies are medically not relevant and can be responsible for the occurrence of adverse effects. (ii) Furthermore, the administration of an antivenom can lead to life-threating systemic effects such as an anaphylactic reaction (IgE or non-IgE mediated). (iii) In addition, antivenoms are costly to produce, often poorly distributed in areas with a high incidence rate and, very often, trained health staff for a good healthcare are lacking.

### 7.2. Supportive Treatment for Management of Local Effects

Although there is a difference between caterpillar species, there seems to be a consensus on the use of the different supportive treatments to treat the local effects. The first aid treatment entails the immediately soft removal of the remaining setae on the skin and to remove the clothes that may be contaminated with the setae as soon as possible. It has been recommended to cover the area with a sticky tape or to use forceps to remove the setae. Clothes are best washed at a minimum of 60 °C to get rid of the setae [88]. After removing the setae, the affected areas need to be washed with soap and water and dried without contacting the skin to prevent crushing and further release of the venom from residual setae (do not use a towel but dry with a hair dryer).

Cutaneous reactions are often treated with oral antihistamines and application of topical corticosteroids. Notwithstanding herewith, it is known that the therapy with antihistamine shows low efficacy and the use of topical corticosteroid is associated with side effects such as skin thinning [4]. However, for many patients, the use of antihistamine or topical corticosteroid is not even enough. Anti-itching products that contain menthol or phenol may help to relieve pruritus [26]. Moreover, in some caterpillars, ice packs or cold-water compresses are used to reduce inflammation, swelling and pain sensation. Also, analgesics such as tramadol hydrochloride may be used [95]. In severe cases of pain, the combination of a local anesthesia with analgesics appear to be a good combination to manage the pain [71]. More rarely, some caterpillars can induce an anaphylactic shock. If this happens, it is recommended to urgently administer epinephrine subcutaneously [3,95].

In the case of contact with the eyes, it is important that the patients urgently consult an ophthalmologist. Often, the eyes are first rinsed thoroughly with water and examined for remaining setae. Topical anesthetic products can assist in examinations and reduce the pain in the eyes that the patients experience. Stinging setae that have penetrated deep into the eye tissue must be removed surgically in order to prevent further damage and irritation [89,96].

Finally, no effective treatment exists to help patients after an accident with the pararama caterpillar, although it is believed that systemic corticosteroids would prevent the onset of a chronic situation wherein joint immobilization, loss of cartilage and bone structure occur [24].

From the above proposed summarization of existing symptomatic and supportive treatments, we can conclude that their availability is quite low and show low efficacy. There are many people who suffer from envenomation by many caterpillars around the globe. This clearly demonstrates the necessity of further research on caterpillars in order to open new perspectives for drug development.

## 8. Future Perspectives

The toxin variation among some caterpillar families or even between species certainly does not make it easy to find a cure that relieves all the symptoms and does not create adverse effects. Therefore, it is important for the future that we extend the extraction of venom components inside urticating setae of different species of Lepidoptera. In the past, researchers mainly focused on the analysis of the venom via the gold standard technique, SDS-PAGE. However, as demonstrated by Berardi et al. (2017) [22], we therefore miss a lot of important information about the venom composition. It is expected that in the future, new tools, such as genomics, transcriptomics and proteomics (LC-MS/MS), that are able to sequence miniscule droplets of the venom, will reveal the presence of much more bioactive molecules or toxins. When more transcriptome and proteome databases become available, it will provide us with essential information on the venom composition of caterpillar species and the inferring function of their proteins. From the new developed genomic databases, bioinformatics tools can be used to search for open reading frames encoding new toxins and possible isoforms. On the other hand, if we really want to unravel and clarify the mechanism and find a cure, we also need to focus on functional studies. At this moment, the research data point to a possible participation of TRPV1 channels and activation of mast cells in provoking the local effects. This of course makes it particularly interesting to investigate other ion channels and receptors, such as other TRP channels, histamine receptors and sodium channels in order to find new target(s) of the toxins in the pathway. This information will enable us to make a correlation between the reactions seen in humans and the identified proteins but also extend it to other Lepidoptera sharing similar defense systems. Only in this way will we get a step closer to discovering and understanding the origin of the complex symptoms and pave the way to new treatments. The ideal treatment would be one that effectively inactivates the toxins, is readily available, easy to use and cheap. There is a great expectation to find a truly selective and potent medicine in the form of a cream, lotion or ointment. Perhaps the search for selective blockers of TRP channels can open new perspectives in finding a treatment to adequately address the conditions. Many side effects can be avoided by using a drug to be applied externally. To conclude, we think that the world of caterpillars has still many unexplored paths to walk. Further research will certainly open new perspectives to combat this 21st-century health hazard.

## 9. Conclusions

Even in the 21st century, caterpillars cause serious health effects, from local dermatitis symptoms to potentially fatal systemic ones. The serious health effects make us realize that, in this modern world of medicine, we urgently need to find a solution. The provoked symptoms are a testimony of a colorful resource of clinically relevant bioactive molecules inside the venom. Recent advances in technologies have provided essential information on the venom protein composition that made it possible to link the effects of the toxins with the symptoms seen in patients. However, there is still a lot of work to do in the world of caterpillar toxins. Understanding the origin of the complex symptoms in the pathogenesis of a caterpillar should pave the way to discovering an effective treatment and this knowledge might be useful to compare and to treat envenomations with caterpillars that share a similar defense mechanism. In this way, the goal shifts to help countless people worldwide.

## Figures and Tables

**Table 1:** Occurrence, cases and venom effects from caterpillars of moths described in this review. The table shows seven of the nine venomous caterpillar families. For the Eucliedae and Nymphalidae family, no reference in the literature was found of envenomation incidences.

Superfamily	Family	Species	Occurrence	Cases		Venom Effects	References
				Year	Number		
Bombycoidea	Saturniidae	*Hylesia metabus*	Venezuela, French, Guiana	-	Epidemic outbreaks Number not defined	Pruriginous dermatitis Edema and Erythema Local tissue damage Pain	[17,30,31,32]
		*Leucanella memusae*	Argentina	-	Not defined	Local tissue damage Pain	[18]
		*Lonomia achelous*	Argentina	-	Not defined	Edema and Erythema Haemostatic disturbances Acute kidney injury Pain	[8,33,34]
		*Lonomia obliqua*	Southern Brazil	2000–2018	60.588 cases 33 mortalities incidence rate of 3,2 envenomations per 100,000 inhabitants	Edema and Erythema Local tissue damage Pain Haemostatic disturbances Acute kidney injury	[8,13,14,15] [16,18,35,36]
Lasiocampoidea	Lasiocampidae	*Dendrolimus pini*	Eastern Europe, Central Asia, Northern Asia, Northern Africa	-	Not defined	Dendrolimiasis: Urticating dermatitis Edema and Erythema Osteoarthritis Pain	[3,4]
Noctuoidea	Erebidae	*Euproctis chrysorrhea*	Japan, China	-	Hundred thousand persons Exact number not defined	Contact dermatitis Edema and Erythema Conjunctivitis Allergic reactions Pain	[4,21]
	Erebidae Subfamily: Arctiidae	*Premolis semirufa*	Brazil	-	Not defined	Local tissue damage Edema and Erythema Pain Joint immobilization Loss of cartilage	[24,34,37,38,39]

**Table 2:** Occurrence, cases and venom effects from caterpillars of moths described in this review. The table shows seven of the nine venomous caterpillar families. For the Eucliedae and Nymphalidae family, no reference in the literature was found of envenomation incidences.

Superfamily	Family	Species	Occurrence	Cases		Venom effects	References
				Year	Number		
Noctuoidea	Notodontidae	*Ochrogaster lunifer*	Australia	-	Endemic to Australia Number not defined	Equine Amnionitis Fetal Loss of horses Contact dermatitis Ophthalmia Severe allergic reaction Pain	[22,25]
	Notodontidae	*Thaumetopoea pityocampa*	Europe: France,Italy,	-	18% of humans infected in outbreak areas 60% of veterinary practitioners in France experience symptoms	Contact dermatitis Edema and Erythema Severe allergic reaction Burning pain Conjunctivitis	[3,21,22,40]
	Subfamily Thaumetopoeinae	*Thaumetopoea processionea*	Belgium, Netherlands, United Kingdom, Germany		Epidemic outbreaks Number not defined		[3,23,40]
Papilionoidea	Nymphalidae	*Morpheis ehrenbergii*	Mexico	-	Not defined	Contact dermatitis Pain	[53]
Zygaenoidea	Megalopygidae	*Megalopyge lanata*	Argentina	-	Not defined	Edema and Erythema Pain	[41]
		*Megalopyge opercularis*	Virginia, Texas	2000–2016	3484 cases	Contact dermatitis Edema and Erythema Local tissue damage Pain	[20]
		*Lagoa crispate*	Oklahoma	-	Not defined	Edema and Erythema Pain	[42]
		*Podalia ca. fuscescens*	Argentina	-	Not defined	Edema and Erythema Pain	[18]
		*Podalia orsilochus*	Argentina	-	Not defined	Edema and Erythema Pain	[18]
	Limacodidae	*Latoia consocia*	China, Taiwan	-	Not defined	Pain	[43]
